# Image Quality and Radiation Dose for Prospectively Triggered Coronary CT Angiography: 128-Slice Single-Source CT versus First-Generation 64-Slice Dual-Source CT

**DOI:** 10.1038/srep34795

**Published:** 2016-10-18

**Authors:** Jin Gu, He-shui Shi, Ping Han, Jie Yu, Gui-na Ma, Sheng Wu

**Affiliations:** 1 Department of Radiology, Union Hospital, Tongji Medical College, Huazhong University of Science and Technology, 1277 Jiefang Avenue, Wuhan 430022, P. R. China

## Abstract

This study sought to compare the image quality and radiation dose of coronary computed tomography angiography (CCTA) from prospectively triggered 128-slice CT (128-MSCT) versus dual-source 64-slice CT (DSCT). The study was approved by the Medical Ethics Committee at Tongji Medical College of Huazhong University of Science and Technology. Eighty consecutive patients with stable heart rates lower than 70 bpm were enrolled. Forty patients were scanned with 128-MSCT, and the other 40 patients were scanned with DSCT. Two radiologists independently assessed the image quality in segments (diameter >1 mm) according to a three-point scale (1: excellent; 2: moderate; 3: insufficient). The CCTA radiation dose was calculated. Eighty patients with 526 segments in the 128-MSCT group and 544 segments in the DSCT group were evaluated. The image quality 1, 2 and 3 scores were 91.6%, 6.9% and 1.5%, respectively, for the 128-MSCT group and 97.6%, 1.7% and 0.7%, respectively, for the DSCT group, and there was a statistically significant inter-group difference (*P* ≤ 0.001). The effective doses were 3.0 mSv in the 128-MSCT group and 4.5 mSv in the DSCT group (*P* ≤ 0.001). Compared with DSCT, CCTA with prospectively triggered 128-MSCT had adequate image quality and a 33.3% lower radiation dose.

Coronary computed tomography angiography (CCTA) has been widely used as a noninvasive technique to evaluate coronary artery lesions, but the carcinogenic potential of high radiation dose is of concern to radiologists, patients, referring physicians, CT scanner manufacturers and regulatory agencies^1^,^2^,^3^.

Several CCTA methods have been used to reduce the radiation dose. The prospectively triggered protocol is a well known scanning technique that can substantially reduce the CCTA radiation dose^4^,^5^, and some studies have indicated that the technique can provide good image quality with a low radiation dose^6^,^7^,^8^,^9^. The second method involves adjusting the scan parameters, such as the tube voltage, tube current and pitch^1^,^10^. Studies have shown that the effective dose increases linearly with the tube current^3^,^11^. The third technique includes iterative reconstruction techniques that can also be used to reduce the radiation dose while maintaining image quality^12^. To reduce the radiation dose, it is important to use different methods with the scanner provided.

Compared with 16-slice and 64-slice CT, 128-slice CT has more detectors, which means larger volume coverage and shorter scan times. These advantages are useful for the application in CCTA. Compared with the other scanner systems^7^,^8^,^13^, 128-slice single-source CT (128-MSCT) can use a low-tube current with acceptable images^14^,^15^. First-generation 64-slice dual-source CT (DSCT) is an advanced scanner for coronary CT angiography with a heart rate independent high temporal resolution of 83 ms. In this study, we compared the image quality and radiation dose of CCTA from prospectively triggered 128-MSCT versus prospectively gated DSCT.

## Materials and Methods

None of the authors in this study are employed by Siemens Medical Solutions (Forchheim, Germany). This study was approved by the Medical Ethics Committee at Tongji Medical College of Huazhong University of Science and Technology. All patients were informed of the purpose of the study, and written consent was obtained from all patients. The study methods were performed in accordance with the approved guidelines.

### Patients

Eighty consecutive patients without revascularization therapy and with stable heart rates lower than 70 bpm before the exam were enrolled in the study. The heart rate change was less than 10 bpm, as recorded before and during the performance of the CCTA. The patients were randomly divided into two groups. Forty patients were scanned with the 128-MSCT (Definition AS+, Siemens Healthcare, Forchheim, Germany), and the other 40 patients were scanned with the DSCT (Somatom Definition, Siemens Healthcare, Forchheim, Germany).

### CT scanning protocols

All patients were scanned with the prospectively triggered sequential scanning mode. The 128-MSCT group was scanned with the following parameters: 128 × 0.6 mm collimation, gantry rotation time 0.3 s, and time resolution 150 ms. The following parameters were used for the DSCT group: 2 × 32 × 0.6 mm collimation, gantry rotation time 0.33 s, and time resolution 83 ms. The tube voltage was 120 kV for both groups, but the reference tube current-time product was 240 mAs for the 128-MSCT group and 400 mAs for the DSCT group. The automatic tube current modulation (ATCM) technique (i.e., CARE Dose 4D in a Siemens scanner) was used to reduce the radiation doses in both groups. The center of the triggering window was set to 70% of the R-R interval, and the padding phase was ± 8% (80 msec) for both groups. The scanning range for all examinations extended from the level of the carina to the diaphragm.

Before the examination, all patients were instructed on breath holding to minimize artifacts during their examinations. Nitroglycerin spray (0.5 mg, Shandong Province, China) was used sublingually 5 min before the examination to dilate the coronary arteries. The contrast material of Iopamidol (400 mgI/ml; Bayer Schering Pharma, Berlin, Germany), followed by 40 ml normal saline, was injected through the antecubital vein with an automatic injector. Bolus tracking was used to start the scan with a tracking area set at the root of the descending aorta. The flow rate was 4.5 ml/s. Because the scan time was shorter in the 128-MSCT group, the patients in this group received only 60–65 ml contrast materials, whereas the patients in the DSCT group received 70–75 ml. There was an additional delay time of 5s before the CT data acquisition.

### Post-processing

The data constructive section thickness was 0.75 mm; the increment was 0.5 mm, and the reconstruction kernel was B26f Heartview smooth. All images were analyzed with semi-automated post-processing software (Aquarius; TeraRecon, San Mateo, CA, USA). Image post-processing methods on the workstation included 3D volume rendering (VR), multi-planar reconstruction (MPR), curved planar reconstruction (CPR), and maximum intensity projection (MIP) using Circulation software.

### Image analysis

In accordance with the scheme proposed by the American Heart Association^16^, the coronary artery tree was divided into 16 segments. The intermediate artery, if present, was designated segment 17. The image quality of the coronary arteries was evaluated for each segment. According to Stolzmann *et al*.^17^, all segments with a diameter of at least 1 mm at their origin were evaluated using a three-point grading scale: score 1 corresponded to excellent image quality (no motion or stair-step artifacts), score 2 indicated moderate image quality (moderate motion artifacts and stair-step artifacts or blurring), and score 3 indicated insufficient image quality (distinct motion artifacts and stair-step artifacts). Images with a score of 1 or 2 were considered to be acceptable for diagnosis. All images were evaluated by two experienced cardiac radiologists who were unaware of the patients’ information. After they performed separate evaluation, the radiologists discussed their findings and agreed on the final image quality.

### Radiation dose

In this study, we calculated the radiation exposure of the CCTA scan. The effective tube current, volume CT dose index and dose-length product of CCTA were obtained from the patient protocol of the system. The effective dose was derived from the product of the dose-length product and a conversion coefficient for the anatomical region examined, as proposed by the Fleischner Society^18^. The conversion coefficient of the chest was 0.017 mSv/(mGy.cm)^3^;

effective dose (mSv) = dose-length product (mGy.cm) × 0.017 mSv/(mGy.cm).

### Statistical analysis

The statistical analysis was performed using SPSS 12.0. Continuous variables were expressed as the mean ± standard deviation (SD), and categorical variables were expressed as frequencies or percentages. The Chi-squared test was used to compare the image quality. Agreement of the image quality scores was assessed with ĸ statistics. The impact of mean heart rate on image quality was assessed by Spearman’s rank-order correlation, with coefficients calculated on a per patient basis. The comparison of the mean radiation dose was performed using an independent-samples t-test. A *P* value < 0.05 was considered statistically significant.

## Results

### Patients

In this study, each group included 40 patients. The patients included asymptomatic patients with cardiac risk factors (29 patients), symptomatic patients with chest pain or stuffiness (to exclude coronary artery lesions, 40 patients) and CAD follow-up without revascularization therapy (11 patients). The patients in the two groups were adequately matched according to age, sex, mean heart rate, and body mass index (*P *= 0.210–0.833, Table 1).

### Scan Parameters

The acquisition times were (4.97 ± 0.58) s in the 128-MSCT group and (8.99 ± 0.98) s in the DSCT group. There was a 44.7% decreased scan time in the 128-MSCT group compared with the DSCT group (*P *≤ 0.001), which was responsible for the lower amount of contrast material and higher triggering threshold in the 128-MSCT group compared with the DSCT group.

The tube voltage was 120 kV in both groups, but on average, the reference tube current was higher for the DSCT scan (400 mAs) compared with the 128-MSCT scan (240 mAs). After the ATCM technique was used, the effective tube current in the 128-MSCT group (196.9 ± 48.2 mAs; range: 93 mAs–285 mAs) was significantly lower than that in the DSCT group (295.7 ± 63.5 mAs; range: 176 mAs–431 mAs) (*P *≤ 0.001).

### Image quality

**Figure 1:**
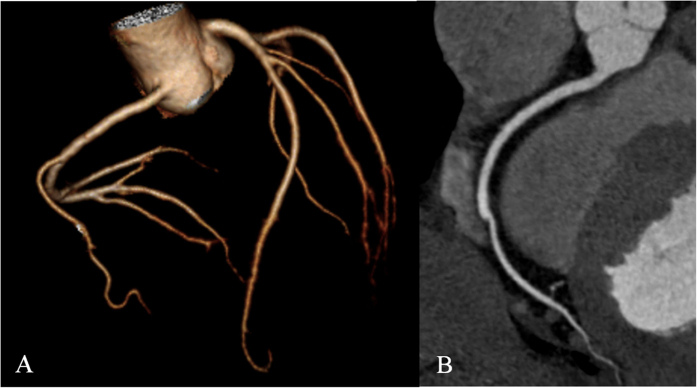
(**A**,**B**) Example of score 1 from the 128-MSCT group: The patient, a 47-year-old man with chest pain, underwent prospectively triggered CCTA using 128-MSCT, and his BMI was 26.4 kg/m^2^. CCTA reconstruction is displayed in 3D VR (**A**) and CPR of RCA (**B**) with good image quality (score 1). The effective dose was 3.6 mSv.

**Figure 2:**
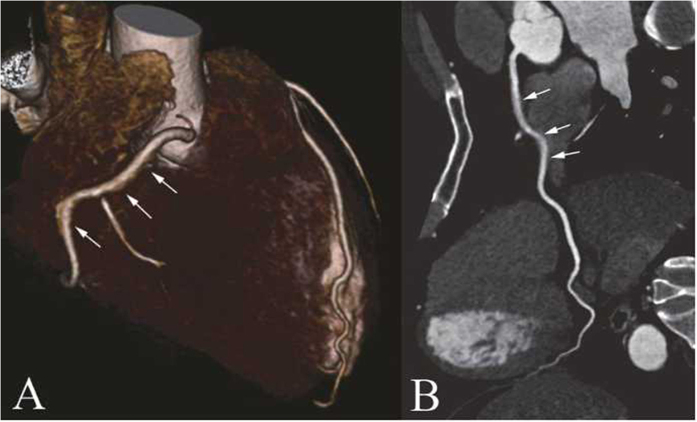
(**A**,**B**) Example of score 2 from the 128-MSCT group: The patient was a 47-year-old man with diabetes who underwent prospectively ECG-gated CCTA, to exclude CAD, with 128-MSCT, and his BMI was 25.0 kg/m^2^. CCTA reconstruction is displayed in 3D VR (**A**) and CPR of RCA. (**B**) The image score of the middle segment of RCA was scored 2 (arrowhead), and the other segments were scored 1. The effective dose was 3.3 mSv.

**Figure 3:**
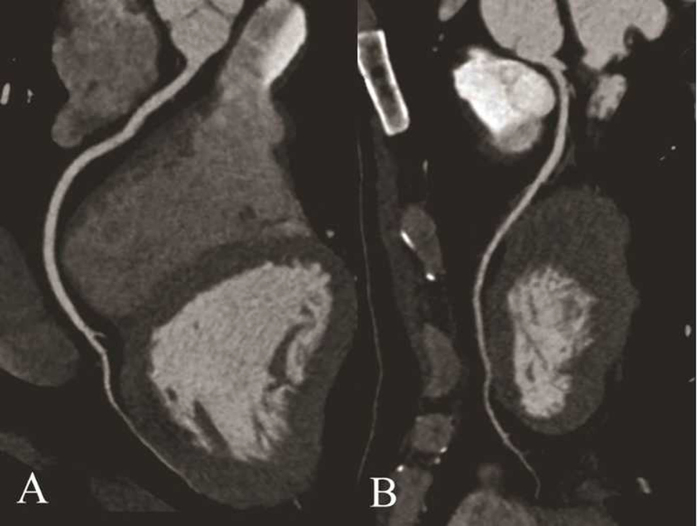
(**A**,**B**) Example of score 1 from the DSCT group: The patient was a 42-year-old man with chest stuffiness who underwent prospectively triggered CCTA, to excluded CAD, with DSCT, and his BMI was 25.2 kg/m^2^. CCTA reconstruction is displayed in CPR for RCA (**A**) and LAD (**B**) with a good image quality (score 1). The effective dose was 4.1 mSv.

Agreement was excellent (ĸ = 0.86) between the two radiologists for the segment image quality scores. In total, there were 1114 coronary arterial segments, and 44 segments were excluded because of their smaller (<1 mm) diameter (25 in the 128-MSCT group and 19 in the DSCT group). The remaining 1070 segments were evaluated (526 segments in the 128-MSCT group and 544 segments in the DSCT group; Table 2). The percentage of segments with diagnosable image quality (scored as 1 and 2) was 98.5% (518/526) for the 128-MSCT group and 99.3% (540/544) for the DSCT group, and the difference was significant (*P *= 0.00012). In the 128-MSCT group, 91.6% (482/526) of the segments were scored as excellent (Fig. 1), but 36 segments, mainly middle segments of the right coronary artery, were evaluated as having moderate image quality (Fig. 2). In the DSCT group, 97.6% (531/544) of the segments were scored as excellent (Fig. 3). The percentage of segments scored as 1 in the DSCT group was significantly higher than in the 128-MSCT group (*P *= 0.001). Eight segments in the 128-MSCT group and 4 segments in the DSCT group had poor image quality and were scored as 3.

### Impact of heart rate and heart rate variability on image quality

**Figure 4:**
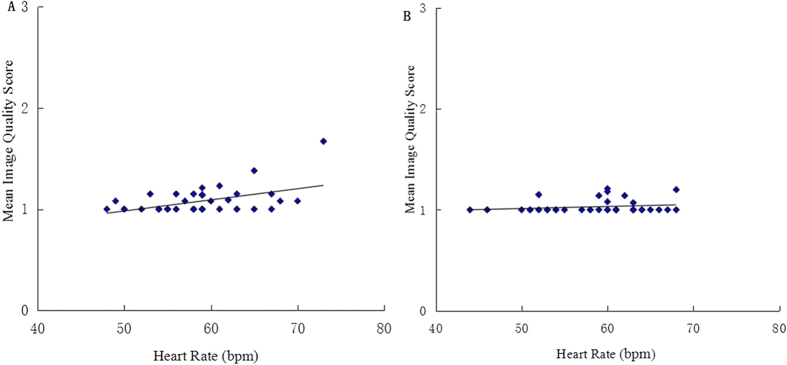
(**A**,**B**) Linear regression plot of mean image quality scores per patient (y-axis) against heart rate (x-axis) by 128-MSCT (**A**) and DSCT (**B**). Spearman’s correlation for the 128-MSCT group (*r *= 0.48, *P *< 0.01); Spearman’s correlation for the DSCT group (*r* = 0.20, *P *= 0.22).

The average heart rate during scanning was 58.6 ± 5.3 bpm (range) for 128-MSCT and 58.1 ± 6.1 bpm (range) for DSCT. A significant correlation between heart rate and mean image quality score was observed by per patient analysis for the 128-MSCT group (*r *= 0.48, *P *< 0.01), whereas no significant correlation was found for the DSCT group (*r *= 0.20, *P *= 0.22), as shown in Fig. 4.

### Radiation dose

The mean effective doses in the 128-MSCT group and DSCT group were 3.0 ± 0.8 mSv (range: 1.3–5.0 mSv) and 4.5 ± 1.0 mSv (range: 2.5–6.7 mSv), respectively. There was a 33.3% radiation dose savings in the 128-MSCT group compared to the DSCT group (t = 7.2, *P *≤ 0.001) (Table 3).

## Discussion

Some studies have compared image quality and radiation dose performed with 128-MSCT, but the comparisons have been performed against retrospective gating protocols and not versus prospective triggering modes^7^,^8^,^14^,^15^. In the present study, we compared image quality and radiation dose performed with 128-MSCT and DSCT (both performed with the prospectively triggered technique). The results suggested that 128-MSCT coronary angiography can be used with the prospective gating technique in patients with stable sinus rhythm and a heart rate below 70 bpm. The radiation dose in the 128-MSCT group was 33.3% lower than that in the DSCT group.

Prospectively triggered CCTA is a sequential technique with no table motion during the beam-on time, and it acquires image data only in a small portion of the cardiac cycle R-R interval. Therefore, this approach significantly reduces the radiation dose, which is the most attractive advantage of this scanning protocol compared to the retrospective ECG-gated technique. Arnoldi *et al*.^7^ have reported a 57–67% dose reduction for 128-MSCT using the prospectively gated technique compared with DSCT using the retrospective ECG-gated technique. Duarte *et al*.^8^ have reported a significant reduction in the radiation dose for 128-MSCT coronary angiography using the prospectively triggered technique compared with 64-slice CT using the retrospective ECG-gated technique.

Coronary CT angiography using the prospectively triggered technique requires high time resolution. In the present study, the image quality of most of the segments in the 128-MSCT group was sufficient for a diagnosis, but there was still a relatively higher rate of moderate image quality and a lower percentage of excellent image quality in the 128-MSCT compared with the DSCT group, primarily in the middle segment of the right coronary artery. This can be explained by several reasons. First, the vertical course of middle segment of right coronary artery which was more sensitive to the cardiac beat than other segments^19^. Budoff *et al*.^4^ have reported that the ballistic movement of mid right coronary artery may be as 5 to 6 times its diameter during the twisting and torsion of the heart during the cardiac cycle, which made the motion artifacts are especially prominent in this segment. Another factor is the relatively lower time resolution of 128-MSCT compared with DSCT (83 ms VS 150 ms). In this study, the average heart rate was good matched in the two groups (128-MSCT: 58.6 ± 5.3 bpm; DSCT: 58.1 ± 6.1 bpm), but a significant correlation was observed for the 128-MSCT group not only between heart rate and mean image quality (*r *= 0.48, *P *< 0.01), which means the heart rate scanned by 128-MSCT should be more strictly controlled to guarantee the image quality and diagnostic accuracy.

The scanning parameters that affect the CT radiation dose include the tube voltage, tube current time product, pitch, section thickness, and scanning length^3^. The radiation dose is linearly related to the tube current when all other factors are fixed^20^,^21^. For the prospectively triggering technique, two methods are used to control the tube current: manual and automatic. The ACTM technique controls the tube current according to the size and attenuation of the body region being scanned throughout the cardiac cycle to decrease the radiation exposure automatically^1^,^22^,^23^. In the present study, after the ATCM technique was applied, the effective tube current was much smaller than the reference tube current. With other scan parameters being the same, there was a 33.3% radiation dose reduction in the 128-MSCT group (3.0 mSv) compared with the DSCT group (4.5 mSv).

Other advantages of 128-MSCT included the wider collimation and shorter acquisition time compared with DSCT. In this study, the acquisition time was 4.97s for the 128-MSCT group and 8.99s for the DSCT group. There was a 44.7% time reduction for the 128-MSCT compared with the DSCT. A shorter acquisition time is beneficial for breath holding, reducing the occurrence of respiratory arrhythmias and the volume of contrast materials and radiation exposure^24^.

This study had some limitations. First, additional dose reduction techniques, such as reducing the tube voltage and the reference tube current time in patients with low body weight, were not applied. Second, most patients did not undergo invasive coronary angiography; therefore, we did not analyze the diagnostic accuracy of the different CT scanners.

## Conclusion

The present study showed that in select patients, coronary CT angiography with prospectively triggered 128-MSCT with a low tube current can provide adequate image quality with a low radiation dose compared with DSCT. We believed that these findings may have clinical utility for patients for whom a lower radiation dose is important, but currently, the technique is limited because it is not recommended at heart rates higher than 70 beats per minute because the time resolution for 128-MSCT is 150 ms.

## Additional Information

**How to cite this article**: Gu, J. *et al*. Image Quality and Radiation Dose for Prospectively Triggered Coronary CT Angiography: 128-Slice Single-Source CT versus First-Generation 64-Slice Dual-Source CT. *Sci. Rep.* **6**, 34795; doi: 10.1038/srep34795 (2016).

## Figures and Tables

**Table 1:** Patient demographics (n = 80; the values are shown as the means ± standard deviations).

Characteristics	128-MSCT	DSCT	*P*
Age (years)	56.2 ± 10.4	58.1 ± 9.2	0.798
Sex [female (male)]	10 (30)	7 (33)	0.833
Mean heart rate (beats/min)	58.6 ± 5.3	58.1 ± 6.1	0.711
Body mass index (BMI, kg/m^2^)	24.5 ± 3.0	25.4 ± 3.4	0.210

There were no significant differences in age, sex, mean heart rate, or body mass index between the two groups (*P *= 0.210–0.833).

**Table 2:** Image quality evaluated with a three-point grading scale (score 1 corresponded to excellent image quality, score 2 to moderate image quality and score 3 to insufficient image quality).

Image quality	128-MSCT	DSCT	*P*
Score 1	482 (91.6%)	531 (97.6%)	≤0.001
Score 2	36 (6.9%)	9 (1.7%)	≤0.001
Score 3	8 (1.5%)	4 (0.7%)	≤0.001

More segments received a score of 1 in the DSCT group than in the 128-slice CT group (*P *= 0.001). More segments with diagnosable image quality (scores 1 and 2) were found in the DSCT group than in the 128-MSCT group (*P *= 0.00012).

**Table 3:** Radiation dose (values are shown as the means ± standard deviations).

Radiation dose	128-MSCT	DSCT	*P*
Volume CT dose index (mGy)	12.0 ± 3.1	19.6 ± 4.5	≤0.001
Dose-length product (mGy.cm)	175.5 ± 48.0	262.2 ± 61.3	≤0.001
Effective dose (mSv)	3.0 ± 0.8	4.5 ± 1.0	≤0.001

*P *≤ 0.01 for comparisons of the volume CT dose index, dose-length product and effective dose between the two groups.
