# Author Correction: Image Quality and Radiation Dose for Prospectively Triggered Coronary CT Angiography: 128-Slice Single-Source CT versus First-Generation 64-Slice Dual-Source CT

**DOI:** 10.1038/s41598-020-68767-9

**Published:** 2020-07-10

**Authors:** Jin Gu, He-shui Shi, Ping Han, Jie Yu, Gui-na Ma, Sheng Wu

**Affiliations:** Department of Radiology, Union Hospital, Tongji Medical College, Huazhong University of Science and Technology, 1277 Jiefang Avenue, Wuhan, 430022 P. R. China

Correction to: *Scientific Reports* 10.1038/srep34795, published online 18 October 2016

This Article contains a typographical error in the Discussion section.

“Another factor is the relatively lower time resolution of 128-MSCT compared with DSCT (83 ms VS 150 ms).”

should read:

“Another factor is the relatively lower time resolution of 128-MSCT compared with DSCT (150 ms VS 83 ms).”

